# Aesthetic Preferences for Eastern and Western Traditional Visual Art: Identity Matters

**DOI:** 10.3389/fpsyg.2016.01596

**Published:** 2016-10-20

**Authors:** Yan Bao, Taoxi Yang, Xiaoxiong Lin, Yuan Fang, Yi Wang, Ernst Pöppel, Quan Lei

**Affiliations:** ^1^School of Psychological and Cognitive Sciences and Beijing Key Laboratory of Behavior and Mental Health, Peking UniversityBeijing, China; ^2^Human Science Center, Institute of Medical Psychology, Ludwig-Maximilians-UniversityMunich, Germany; ^3^Parmenides Center for Art and SciencePullach, Germany; ^4^Department of Psychology, University of Minnesota, MinneapolisMN, USA

**Keywords:** beauty, culture, aesthetics, visual perception, Chinese painting, Western painting

## Abstract

Western and Chinese artists have different traditions in representing the world in their paintings. While Western artists start since the Renaissance to represent the world with a central perspective and focus on salient objects in a scene, Chinese artists concentrate on context information in their paintings, mainly before the mid-19th century. We investigated whether the different typical representations influence the aesthetic preference for traditional Chinese and Western paintings in the different cultural groups. Traditional Chinese and Western paintings were presented randomly for an aesthetic evaluation to Chinese and Western participants. Both Chinese and Western paintings included two categories: landscapes and people in different scenes. Results showed a significant interaction between the source of the painting and the cultural group. For Chinese and Western paintings, a reversed pattern of aesthetic preference was observed: while Chinese participants gave higher aesthetic scores to traditional Chinese paintings than to Western paintings, Western participants tended to give higher aesthetic scores to traditional Western paintings than to Chinese paintings. We interpret this observation as indicator that personal identity is supported and enriched within cultural belongingness. Another important finding was that landscapes were more preferable than people in a scene across different cultural groups indicating a universal principle of preferences for landscapes. Thus, our results suggest that, on the one hand, the way that artists represent the world in their paintings influences the way that culturally embedded viewers perceive and appreciate paintings, but on the other hand, independent of the cultural background, anthropological universals are disclosed by the preference of landscapes.

## Introduction

The concept of beauty is a complex topic since antiquity, and this is especially true when tracing the cultural trajectory of our relationship with beauty. Western and Eastern artists tend for instance to use different perspectives to represent the visual world, both in the geometric and in a metaphorical sense. Viewers from different cultures and social groups may have distinct aesthetic experiences to the same visual displays (Palmer et al., 2013). Cultural differences might explain why beauty is attributed to some things, but not to others (Jacobsen, 2010). Aesthetic processing can only be understood, if it is also seen as being embedded in cultural contexts and being modulated by social conditions.

Unlike Western painters who since the Renaissance tried to create an exact view of a visual environment, Chinese painters never developed a notion of space as a measurable geometrical entity by developing mathematical rules to organize space and create precise spatial relations (Delahaye, 1993). Instead, the Chinese outlook emphasizes a dynamic structure for human relations with the environment, even with the universe, independent of exact physical representations or the proper imitation of objects (Sullivan, 1984; Cameron, 1993). Pictorial perspectives employed in Western and Chinese paintings are, thus, fundamentally different. Western painters tried to create an exact view of what they see (or what they believe to see); the geometric perspective was developed to create the illusion of three-dimensionality by means of a single-point or convergent perspective (Kubovy, 1986). It should, however, be pointed out that the central perspective in Western art is already an abstraction (Worringer, 1908), and it is not at all a geometrically correct representation of what we see. Mechanisms of size constancy (Pöppel, 1988) recalibrate the projection of visual stimuli on the retina at the cortical level, and thus distort what is mathematically defined. This neural operation in the early visual pathway (Zhou et al., 2016) serves the purpose to maintain the identity of the perceived object. Thus, the different trajectories of abstraction in the Eastern and Western cultural environments have created unique conceptual frames.

Chinese painters have employed specific ways of emphasizing spatial information compared to Western painters. Besides a typical arrangement of spatial information in a vertical manner (i.e., far objects appear in the upper part while close objects appear in the lower part of a scroll painting), a most common means of suggesting distance was perhaps the use of a perspective, where parallel diagonal lines strike off from the plane of the picture. The distinctive characteristics of parallel projections is that lines parallel in fact are also parallel in the drawing. The angles of these obliques are coherent throughout the plane (Tyler and Chen, 2011). Moreover, Western artists are inclined to capture a specific moment in a visual scene and fix the physical position of the viewer. In contrast, when looking at a Chinese landscape painting, there is no distinct point to guide viewers. The Chinese outlook has a dynamic quality that integrates successive time windows (Bao et al., 2015), and encompasses a panoramic view of the visual scene, which can be perhaps associated with a floating view (Tyler and Chen, 2011).

Another concept with respect to differences between Eastern and Western landscapes (Pöppel, 2006) distinguishes on the psychological level between an internal view (“Ich-Nähe” in German) and an external view (“Ich-Ferne” in German); (it should be mentioned in passing that in this area of research many publications are available in other languages that remain mute for the only English-speaking scientific community). The central perspective in Western art (with its misunderstood geometrical law) represents an external point of view, and it is characterized by its own aesthetic values; the visual world is expanding in front of the eyes of the viewer (Ich-Ferne). Other than implied by Masuda et al. (2008) who refer to this view as “insider perspective,” we characterize this external view as “Ich-Ferne.” In Eastern landscapes a completely different psychological mechanism is initiated when viewing a picture from a floating perspective. Because of the multi-layer viewpoints on top of each other on a scroll form, the spectator has the impression being invited to shift one’s position dynamically, sometimes being located in the air (e.g., looking downward from above), sometimes being located on the ground (e.g., looking at scenes straight ahead), and sometimes being located at a lower land (e.g., looking upward at faraway mountains); much more importantly, however, is the psychological consequence of this shifting position that the viewer becomes subjectively a part of the scene. The multi-layer perspectives can be considered to simulate a three-dimensional space resulting in a virtual circle or ellipse vertical to the picture; within this imaginary circle or ellipse the viewer becomes part of the scene depicted in front of the eyes. This implicit construction of subjective space creates the feeling of belongingness or “Ich-Nähe.” Thus, we want to submit that the floating perspective does not represent an “outsider perspective” (Masuda et al., 2008).

Another interesting difference with respect to perspective in a more general sense is related to the pictorial subjects of Western and Chinese paintings. Western artists favor object-centered scenes, whereas Chinese artists prefer context-oriented scenes. Paintings in the West typically seek to make the object salient, i.e., to distinguish the object from the background (Masuda et al., 2008). In China it has been otherwise; Chinese artists put great emphasis on the context, often with a meditative theme showing small human figures, as if humans are embedded in a natural environment and awed or inspired by a mountainous landscape (Turner, 2009), or even overwhelmed by the sublime (Burke, 1757).

Previous research on culture and aesthetics has demonstrated indeed substantial cultural variations in artistic expressions, such as in drawings, photography, city design, product design, or else (for a review, see Masuda et al., 2012). By analyzing the ratio of the horizon drawn to the frame and the number of objects used in 15th to 19th century paintings from East Asian and Western countries, Masuda et al. (2008) provided evidence showing that the East Asian artists placed horizon lines higher than Western artists, and that the size of models in East Asian masterpieces was smaller than that in Western ones. Furthermore, this cultural variation in artistic expressions persisted in landscape drawings of contemporary adult members of North American and East Asian cultures. This pioneer study and subsequent research (Wang et al., 2012; Ishii et al., 2014; Nand et al., 2014; Senzaki et al., 2014) have shown that cultural variations in artistic expressions are empirically testable and robust from a methodological point of view.

However, with respect to this methodological point, another critical factor has to be considered when comparing artifacts from different cultures. According to the theory of mutual constitution between culture and the mind (Shweder, 1991; Morling and Lamoreaux, 2008), people should prefer artistic expressions which reflect their own cultural systems. This prediction is based on the idea that people who are exposed to different types of cultural artworks could internalize their preference for them. To date, several studies have documented cultural influences on a wide range of psychological processes, notably attention, motivation, reasoning and self-concept (Markus and Kitayama, 1991; Nisbett et al., 2001; Han and Northoff, 2009).

In spite of the vast knowledge already gathered (e.g., Masuda et al., 2008; Ishii et al., 2014; Senzaki et al., 2014), we believe that it is still useful to look at one more detail when comparing Eastern and Western art, and possibly evaluating the results within a different frame of reference. Thus, the present study addresses one central question: Are different representations as expressed in typical traditional Chinese and Western paintings appreciated differently by people from different cultural groups? To answer this question, we explored the possibility of cultural differences in aesthetic preferences of contemporary members from the two cultural groups: Chinese and Westerners. We hypothesized that Western and Chinese subjects would show distinct aesthetic preferences due to the implicit application of cultural patterns of artistic expression from their own cultures. This hypothesis on “cultural imprinting” is in line with previous observations (Bao et al., 2013b, 2014) in which it was shown that the language environment shapes temporal processing when a tonal and a non-tonal language are compared; this process is suspected to take place on an implicit level by informal learning (Pöppel and Bao, 2011). It is furthermore suggested that the analytic and holistic strategies are employed also in cognitive processes when representatives from the Eastern and Western cultures evaluate visual artwork validating previous work (e.g., Masuda et al., 2008).

## Materials and Methods

### Participants

Forty-six university students (23 Chinese and 23 international students from Western countries) participated in the experiment. The Western students were from US, Canada and Europe with 15 males and 8 females. They were aged from 18 to 31 years old with an averaged age of 23.74 years. None of the Western participants had lived in China for more than 4 years. The Chinese subjects consisted of 9 males and 14 females, aged from 19 to 30 years old with an averaged age of 23.35 years. All participants had normal or corrected-to-normal visual acuity and color vision, were right-handed, and had no history of neurological disease. None of them were specialists in art history or art theory. Participants were asked before the experiment about their preference on painting style. They generally did not show any specific interest in a certain painting style. All subjects were given informed written consent before the experiment. The study was approved by the departmental ethical committee of Peking University.

### Apparatus

The experiment was conducted in a dimly illuminated room to reduce visual distraction. Picture presentation was controlled by the E-prime software system (Schneider et al., 2002a,b) and displayed on a 19-in CRT monitor (1024^∗^768 resolution, 100 Hz refresh rate). Responses were collected through a keyboard.

### Materials

Sixty traditional Chinese paintings and 60 Western classicist paintings were selected from the archives of http://www.artcyclopedia.com and http://www.namoc.org by the authors in consultation with an art specialist. Both Chinese and Western paintings included two categories, namely, landscapes, and people in a scene. The category “landscapes” refers to depictions that treat nature as the primary topic, and mainly includes sky, mountains, rivers, trees, flowers, meadows, houses, and boats. The category “people in a scene” depicts more than one person engaged in activities, coexisting with backgrounds of the land, thus distinguishing it from portraits. The paintings were chosen from a variety of historical periods (from the 9th to the 18th century). We trust to have selected an appropriate sample of pictures, but we are aware of the fact that some hidden bias may have remained uncontrolled; one has to acknowledge that it is impossible to draw in a statistical sense a “true” random sample from artwork, because the population from which to draw the sample is not definable due to the cultural and historical complexity. In spite of these constraints we believe to have chosen a fair sample of typical pictures from the two cultural environments. To come closer to the goal of an appropriate comparison, all paintings were low in emotional intensity, that is, they did not depict sexual, aggressive, or religious themes. All paintings were prepared in uncompressed bitmap file format, and the image dimensions varied. Graphic manipulation of stimuli was done using Photoshop (Adobe). Each combination of cultural style (Chinese vs. Western Painting) × pictorial subject (landscape vs. people in a scene) includes 30 images. Another 40 images (with 10 images in each condition) were selected from the same database (from which the images for the main experiment were selected) and used in the practice session before the main experiment.

### Procedure

All paintings were presented in random order. Each picture was presented once during the experiment. After viewing each picture subjects were asked to judge its beauty on an 8-Point Scale by pressing one of eight buttons on a keyboard, where 1 indicated very ugly and 8 indicated very beautiful. We also recorded reaction time (RT), but stimulus presentation was self-paced and participants were instructed to approach the paintings in a subjective and engaged manner. Before the main experimental trials, subjects were given 10 practice trials under each condition so they could establish a general impression of the stimuli to be presented. The images used in the practice trials were not used in the experiment.

## Results

**FIGURE 1:**
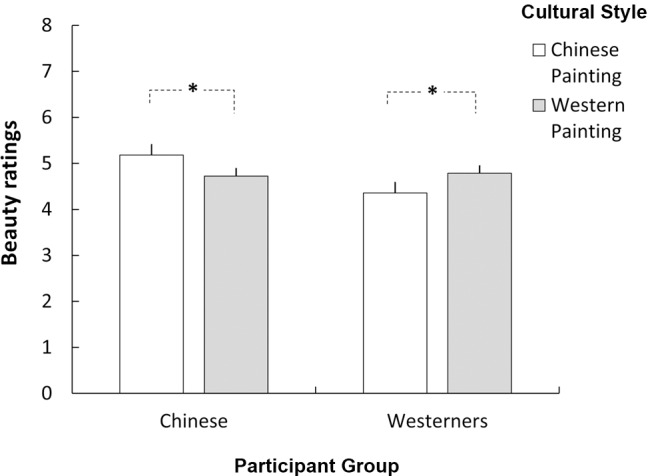
**The significant interaction between Cultural Style (Chinese vs. Western Painting) and Participant Group (Chinese vs. Westerners) on beauty rating.** Chinese and Western participants showed preferences for their own culture’s paintings: Chinese participants gave higher aesthetic scores to traditional Chinese paintings than Western paintings, whereas Western participants did the opposite. ^∗^ *p* < 0.05.

The beauty-rating data were subjected to a three-way mixed analysis of variance (ANOVA) with Cultural Style (Chinese vs. Western Painting) and Pictorial Subject (landscape vs. people in a scene) as two within-subjects variables and Participant Group (Chinese vs. Westerner) as one between-subjects variable. The ANOVA revealed a significant interaction between Participant Group and Cultural Style {[*F*(1,44) = 9.247, *p* < 0.01, η_p_^2^ = 0.174]}, while both main effects of Participant Group and Cultural Style were not significant {[*F*(1,44) = 2.597, *p* = 0.114, η_p_^2^ = 0.056] and [*F*(1,44) = 0.010, *p* = 0.919, η_p_^2^ = 0.000] respectively}. Further analysis of this interaction displayed interesting beauty-rating patterns between the two participant groups: for the Chinese group, a significantly higher score was observed for Chinese paintings relative to Western paintings (5.18 vs. 4.72, *p* < 0.05). For the Westerner group, a reversed pattern was observed, i.e., a significantly higher score was demonstrated for Western painting as compared to Chinese painting (4.78 vs. 4.36, *p* < 0.05) (**Figure 1**). This double dissociation result pattern suggests that Chinese and Western participants prefer paintings that correspond to the background within which they were culturally imprinted.

**FIGURE 2:**
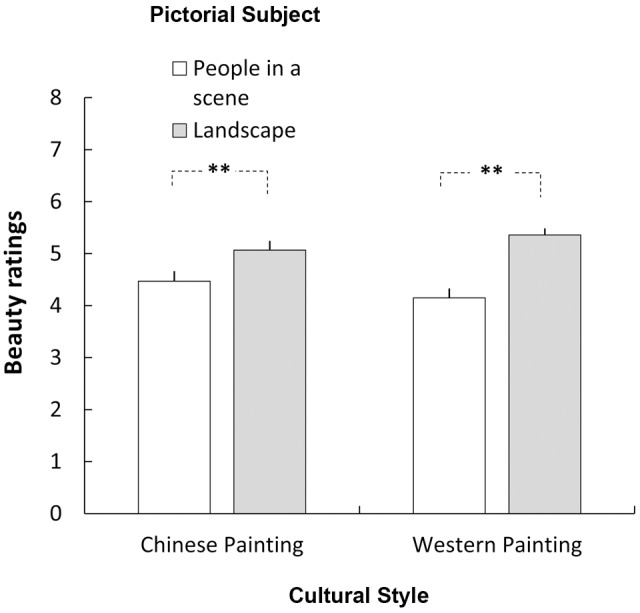
**Beauty rating of paintings as a function of Cultural Style (Chinese vs. Western Painting) and Pictorial Subject (landscape vs. people in a scene).** Both Chinese and Western participants gave higher aesthetic scores to landscape than the people in a scene. ^∗∗^ *p* < 0.01.

**FIGURE 3:**
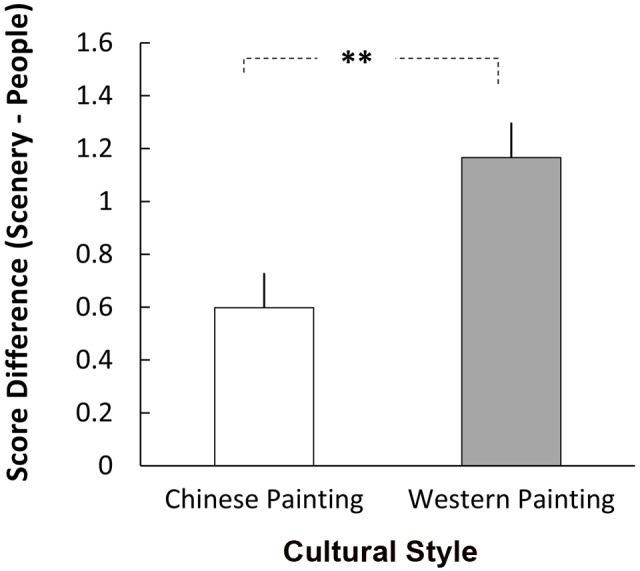
**The difference in aesthetic scores (landscape – people in a scene) was significantly larger for Western paintings than that of Chinese paintings.** ^∗∗^ *p* < 0.01.

The ANOVA produced only one significant main effect for the Pictorial Subject [*F*(1,44) = 37.478, *p* < 0.001, η_p_^2^ = 0.502]; this factor interacted with Cultural Style [*F*(1,44) = 19.338, *p* < 0.001, η_p_^2^ = 0.305]. For both Chinese and Western paintings, participants gave higher scores to landscape than to the category “people in a scene” (**Figure 2**). Further analysis revealed that the difference in scores between Western landscape and figure paintings was significantly larger than that for the Chinese ones (1.20 vs. 0.60, *p* < 0.001) (**Figure 3**). No other main effects or two-way interaction reached significant level. The three-way interaction was also not significant [*F*(1,44) = 0.549, *p* = 0.463, η_p_^2^ = 0.012].

## Discussion

Research in the past has shown that by using stimuli from the arts, i.e., from music, poetry or visual arts, one can obtain new insight into cognitive mechanisms which may remain undetected if one focuses only on simple stimulus configurations as have been employed in the tradition of classical psychophysics (e.g., from our own research environment: Silveira et al., 2012; Avram et al., 2013; Lutz et al., 2013; Pöppel et al., 2013; Zaytseva et al., 2014; Park et al., 2015). With the study reported here, we want to further contribute to this research paradigm by comparing the appreciation of art in subjects from the East and the West with its challenging differences (Pöppel and Bao, 2016). The present study investigated aesthetic preferences of two cultural groups using pictorial representations from the different cultures as stimuli. Our results showed that subjects prefer paintings that correspond to their own cultural traditions, i.e., each cultural group evaluated the paintings from their own culture as more beautiful.

This result at first sight might not at all be surprising as it might simply reflect the well-known “in-group bias” or “in-group favoritism” effect (e.g., Tajfel et al., 1971). One could argue that the subjects immediately recognize whether they are confronted with a picture from the East or from the West, and Eastern subjects feel more familiar with pictures from their cultural background whereas the contrary is true for the Western subjects. If the in-group bias applies in this case, one has to add, however, further arguments, which explain the direction of the bias, because such a bias cannot be anticipated with respect to “aesthetic evaluation.” In the case that Eastern subjects would have evaluated Western pictures as more beautiful, and Western subjects would have preferred Eastern pictures (which also could have happened), one would also deal with in-group bias, but with a reversed direction. Thus, it is necessary to find a reason for the direction of the observed bias in our study. With respect to this question we want to return to one hypothesis outlined above that Eastern and Western pictures create a different psychological state of involvement or “belongingness“ (Ich-Nähe vs. Ich-Ferne). It is argued that the pictures trigger a culturally specific feeling of identity (Pöppel, 2010). A Western subject looking at a Western picture is supported in his feeling of cultural identity, and the same is true for an Eastern subject when looking at an Eastern picture. We want to submit that the creation and maintenance of identity is one of the most fundamental challenges of the human mind (Zhou et al., 2014), and artwork of one’s own cultures may serve as an important psychological mechanism.

Our analysis may be supported by a recent study in which it was reported that when viewing traditional Chinese landscape paintings, Chinese subjects experienced a greater level of relaxation and mind-wandering, and a lower level of object-oriented absorption than when viewing Western realistic landscape paintings (Wang et al., 2014). With respect to cultural identity, the study by Masuda et al. (2008) may also support our viewpoint; they reported that East Asian subjects were more likely to include great details and background when drawing a scene or taking photographs of a model compared to Western subjects.

Some further points have to be appreciated: It has been argued that Westerners apply more rational or logical methods to a wide range of intellectual and artistic pursuits, in which a mathematical orientation plays an important role (Kline, 1964). Western paintings, hence, emphasize the creation of realistic scenes as much as possible. In contrast, Chinese artists place more faith on intuitive and aesthetic knowledge about nature (Golas, 2014). This faith is bolstered by considerable reliance on personal feelings and emotions embedded into the image, rather than the details and exact appearance provided by sensory modalities. Members of different cultural groups are repeatedly exposed to various examples of visual images from their respective cultures, and they may implicitly gain knowledge (Pöppel and Bao, 2011) about the dominant aesthetic representation of the world; thus, the appreciation of paintings that obey aesthetic principles within their culture is facilitated.

Consistent with Shweder’s (1991) argument that psychological processes and cultural products represent two sides of the same coin, Morling and Lamoreaux (2008) further suggested that culture and the mind are mutually constructed. A given cultural meaning system is internalized by members of the culture, and those who internalize that system display habitual ways of thinking and acting. A recent study by Ishii et al. (2014) showed that European Americans preferred unique colorings and Japanese preferred harmonious colorings, and these preferences were positively associated with cultural values, i.e., uniqueness among European Americans and harmony among Japanese participants. Another study (Wang et al., 2012) found that East Asians were more likely than their European Canadian counterparts to prefer the moderately complex webpage to the simple portal page, and the results could be explained by the fact that the Western way of thinking is more self-contained and independent, while most East Asians are more holistic and context oriented. These previous findings, combined with the present results, provide supportive evidence that people indeed prefer artistic expressions which reflect dominant cultural meaning systems.

A surprising result in our study is that both Western and Chinese subjects prefer landscapes compared to the category “people in a scene.” This observation suggests that in spite of the cultural frame of aesthetic appreciation as noted above there may exist an overriding principle with respect to the sense of beauty reflecting an anthropological universal (Bao and Pöppel, 2012). Such an overriding principle at a lower perceptual level is for instance observed in color preferences. Komar and Melamid systematically examined the artistic preferences of people in ten countries, and found that the most preferred painting was an idealized blue landscape (Wypijewski, 1997). There is indeed evidence that color preferences are universal across cultures (e.g., Eysenck, 1941), although later research revealed that both similarities and differences may exist (Taylor et al., 2013). A strong case, however, for a universal color preference has been made for blue (Saito, 1996; Ou et al., 2004).

From the viewpoint of Darwinian aesthetics (or “evolutionary aesthetics”), it has been suggested that humans may be biologically primed to find particular features more beautiful, because these features may have been selected for optimal survival, for instance allowing better decisions about when to move, and where to settle, and what activities to engage in (Thomhill, 1998; Zaidel, 2010). However, evolutionary theorists have been criticized for regarding art only with respect to adaptive preferences (Plotkin, 2004). Apart from ultimate adaptive valence, we are given no criteria by evolutionary aesthetics theories for explaining why some objects are generally perceived as aesthetically superior. Here we suggest that the present finding that landscape is aesthetically more appreciated is not only because it signals restfulness or safety, but also because its restful or safe features carry added emotional significance.

It is worth noting that the difference between the preferences of landscape and people in a scene was higher for Western paintings compared to Chinese paintings. The aesthetic basis of Chinese paintings is deeply affected by the philosophy of Chinese Taoist ideas that emphasize the harmonious relationship between human beings and the cosmos (Law, 2011). In the eyes of Chinese artists, natural scenes have the power to suggest the very essence of life to human beings, and in unobtrusive ways, may therefore act as inspirations to virtue. Indeed, in Chinese landscape paintings we can find tiny human figures, such as a fisherman on a lonely boat, a man following a mountain path, or a man meditating in a cottage. Here the relationship between man and the natural world is the reverse of the case of Western paintings. Thus, one possible explanation for the smaller difference in the preferences of Chinese paintings is that Chinese landscape paintings are focusing on the natural scenes with human figures embedded, although small and not very prominent optically, whereas in Western landscape pictures this is rarely the case.

One important aspect which should not be overlooked is the fact that pictures in both cultures elicit the attention of the viewer. In this case we are confronted with a surprising paradox which mainly applies to Western pictures. With the central perspective in landscape paintings a wide area of the environment is represented which in reality would cover the entire visual field. In the picture, however, the visual angle is much smaller being limited to the perifoveal region. It has been shown, however, that attentional control is different for the perifoveal region and the periphery of the visual field (Bao and Pöppel, 2007); this eccentricity effect of attentional control has been well documented with a number of different experimental paradigms (e.g., Lei et al., 2012; Bao et al., 2013a). Given this situation we are confronted with a paradox: What corresponds to the visual environment in reality, and triggers the two different attentional systems, is contracted in a picture into a much smaller visual representation. This spatial contraction results in a mismatch between the natural perceptual process and its pictorial representation. What should represent physical reality, does not do it at all. On the basis of this paradoxical situation we submit the hypothesis that such a mismatch by itself leads to an external point of view. It enforces “Ich-Ferne” as this artificial perspective does not match reality. The viewer has to deal with an abstraction in the pictorial representation as has been pointed out a long time ago by Worringer (1908). Quite the contrary, the floating perspective in Eastern pictures supports “Ich-Nähe,” and belongingness or embeddedness as indicated above. These different perspectives in a general sense also correlate with different cognitive strategies. The more analytical strategy corresponds to the external point of view, as the viewer is forced to take a position from the distance; the more holistic approach as has been pointed out previously (Masuda et al., 2008; Senzaki et al., 2014) is typical for the Eastern perspective, and as we want to submit being the consequence of the feeling of belongingness and the validation of personal identity. It is interesting to note that such different cognitive strategies have also been observed on a very basic level in auditory processing (Bao et al., 2013b).

Taken together, our study shows both cultural specifics and anthropological universals. Different perspectives presented in traditional Chinese and Western paintings are appreciated differently by Chinese and Westerners, showing a cultural difference in aesthetic preference. The way that artists represent the visual world in their paintings influences the way that viewers perceive their paintings. We suggest that the cultural difference in aesthetic preference is correlated with cultural and social practices in everyday life. Our aesthetic sense is to some extent modulated by the cultural environment in which we grow up. At the same time, however, results in this study indicate an overriding principle that independent of the cultural background pictorial representations of landscapes compared to people have a higher aesthetic value.

## Author Contributions

Study conception and design: YB and EP. Acquisition of data: QL, YF, and YW. Analysis and discussion of data: QL, TY, and XL. Drafting of manuscript: YB and TY. Critical revision: EP, YB, and QL.

## Conflict of Interest Statement

The authors declare that the research was conducted in the absence of any commercial or financial relationships that could be construed as a potential conflict of interest.
